# Trans-ethnic gut microbiota signatures of type 2 diabetes in Denmark and India

**DOI:** 10.1186/s13073-021-00856-4

**Published:** 2021-03-03

**Authors:** Camila Alvarez-Silva, Alireza Kashani, Tue Haldor Hansen, Nishal Kumar Pinna, Ranjit Mohan Anjana, Anirban Dutta, Shruti Saxena, Julie Støy, Ulla Kampmann, Trine Nielsen, Torben Jørgensen, Visvanathan Gnanaprakash, Rameshkumar Gnanavadivel, Aswath Sukumaran, Coimbatore Subramanian Shanthi Rani, Kristine Færch, Venkatesan Radha, Muthuswamy Balasubramanyam, Gopinath Balakrish Nair, Bhabatosh Das, Henrik Vestergaard, Torben Hansen, Sharmila Shekhar Mande, Viswanathan Mohan, Manimozhiyan Arumugam, Oluf Pedersen

**Affiliations:** 1 Novo Nordisk Foundation Center for Basic Metabolic Research, University of Copenhagen, Blegdamsvej 3B, DK-2200 Copenhagen N, Denmark; 2 Danish Academy of Diabetes, Odense University Hospital, DK-5000 Odense C, Kløvervænget 6, Odense, Denmark; 3 Department of Cardiology and Endocrinology, Slagelse Hospital, Slagelse, Denmark; 4 TCS Research, Tata Consultancy Services Limited, 54B Hadapsar Industrial Estate, Pune, 411013 India; 5 Madras Diabetes Research Foundation, No. 4, Conran Smith Road, Gopalapuram, Chennai, 600 086 India; 6 Molecular Genetics Laboratory, Infections and Immunology, Translational Health Science and Technology Institute, NCR Biotech Science Cluster, Faridabad, 121001 India; 7 Steno Diabetes Center Aarhus, Aarhus Universitetshospital, Hedeager 3, 2. sal, Aarhus, 8200 Denmark; 8 Center for Clinical Research and Prevention, Bispebjerg and Frederiksberg Hospitals, University of Copenhagen, Copenhagen, Denmark; 9 Faculty of Health Sciences, University of Southern Denmark, Odense, Denmark

**Keywords:** Gut microbiota, Trans-ethnic, Indians, Danes, Populations, Type 2 diabetes, Metformin

## Abstract

**Background:**

Type 2 diabetes (T2D), a multifactorial disease influenced by host genetics and environmental factors, is the most common endocrine disease. Several studies have shown that the gut microbiota as a close-up environmental mediator influences host physiology including metabolism. The aim of the present study is to examine the compositional and functional potential of the gut microbiota across individuals from Denmark and South India with a focus on T2D. Many earlier studies have investigated the microbiome aspects of T2D, and it has also been anticipated that such microbial associations would be dependent on diet and ethnic origin. However, there has been no large scale trans-ethnic microbiome study earlier in this direction aimed at evaluating any “universal” microbiome signature of T2D.

**Methods:**

16S ribosomal RNA gene amplicon sequencing was performed on stool samples from 279 Danish and 294 Indian study participants. Any differences between the gut microbiota of both populations were explored using diversity measures and negative binomial Wald tests. Study samples were stratified to discover global and country-specific microbial signatures for T2D and treatment with the anti-hyperglycemic drug, metformin. To identify taxonomical and functional signatures of the gut microbiota for T2D and metformin treatment, we used alpha and beta diversity measures and differential abundances analysis, comparing metformin-naive T2D patients, metformin-treated T2D patients, and normoglycemic individuals.

**Results:**

Overall, the gut microbial communities of Danes and Indians are compositionally very different. By analyzing the combined study materials, we identify microbial taxonomic and functional signatures for T2D and metformin treatment. T2D patients have an increased relative abundance of two operational taxonomic units (OTUs) from the *Lachnospiraceae* family, and a decreased abundance of *Subdoligranulum and Butyricicoccus*. Studying each population per se*,* we identified T2D-related microbial changes at the taxonomic level within the Danish population only.

Alpha diversity indices show that there is no significant difference between normoglycemic individuals and metformin-naive T2D patients, whereas microbial richness is significantly decreased in metformin-treated T2D patients compared to metformin-naive T2D patients and normoglycemic individuals. Enrichment of two OTUs from *Bacteroides* and depletion of *Faecalibacterium* constitute a trans-ethnic signature of metformin treatment.

**Conclusions:**

We demonstrate major compositional differences of the gut microbiota between Danish and South Indian individuals, some of which may relate to differences in ethnicity, lifestyle, and demography. By comparing metformin-naive T2D patients and normoglycemic individuals, we identify T2D-related microbiota changes in the Danish and Indian study samples. In the present trans-ethnic study, we confirm that metformin changes the taxonomic profile and functional potential of the gut microbiota.

**Supplementary Information:**

The online version contains supplementary material available at 10.1186/s13073-021-00856-4.

## Background

Type 2 diabetes (T2D) is a chronic metabolic disease characterized by elevated blood glucose levels, primarily caused by impaired insulin secretion, increased hepatic glucose production, and insulin resistance [1]. T2D represents a major global health challenge, affecting an estimated 382 million people worldwide, with an expectation to reach 592 million by 2035 [2]. Although genetics, sedentary life, overeating and other environmental exposures throughout life are well-known risk factors of T2D, recently several studies have shown that an aberrant gut microbiota is a characteristic feature of the disease [3–5]. However, several discrepancies have been reported between studies, mainly due to multiple confounding factors including ethnic differences, lifestyle, prescribed pharmacotherapy and technical variations in procedures for examining the gut microbiota [6].

Metformin, an anti-hyperglycemic agent, the therapeutic effect of which includes a reduction of hepatic glucose production and an increase of insulin sensitivity, is the most commonly prescribed drug for the treatment of T2D [7]. Some evidence indicates that a part of the metformin effect on glucose metabolism may be mediated by intestinal mechanisms including the gut microbiota. For example, intravenous administration of metformin does not have the same glucose-lowering effect compared to oral administration [5, 7], indicating that intestinal and potentially microbiota-mediated mechanisms may be part of the blood glucose-lowering effects of the drug [4].

In a Chinese-Scandinavian study, Forslund et al. [4] showed country-specific microbial signatures for T2D and for metformin treatment. It was demonstrated that demographic variation and metformin treatment were important confounding factors in T2D microbiota studies. Here, we have performed a trans-ethnic study with the aim to characterize the gut microbiota in Indian and Danish adults in the context of T2D. We included 279 Danish volunteers (138 normoglycemic (NG) individuals and 141 with T2D) and 294 South Indian volunteers (137 NG individuals and 157 with T2D). We show major compositional differences of the gut microbiota between Danish and South Indian individuals, some of which may relate to differences in ethnicity, lifestyle, and demography. In addition, comparing the combined group of T2D patients and NG individuals from the two countries, we find gut microbial signatures (taxonomic and predicted functional potential signatures) of T2D and metformin treatment, respectively.

## Methods

### Study design and sample collection

To minimize the confounding effects of the technical procedures, we (i) synchronized our standard operating procedures for recruitment of study participants, biological sample processing, and microbial DNA extraction of stools and (ii) performed nucleotide sequencing of all samples in one sequencing center. Similarly, profiling of inflammation biomarkers from all samples was also performed in the same laboratory.

#### Danish sub-study

Three hundred and eight Danish volunteers were recruited from outpatient clinics at Steno Diabetes Center (Gentofte, Denmark), Herlev Hospital (Herlev, Denmark), and Aarhus University Hospital (Aarhus, Denmark); recruited as part of DanFund [8] and ADDITION-PRO [9] cohorts; and recruited by advertisement in local newspapers. All Danish individuals were of White European ethnicity, aged 35 to 74 years, with a body mass index (BMI) from 20 to 40 kg/m^2^. Individuals who were treated with antibiotics within 4 months, who were pregnant or lactating, or who were unable to give informed consent were ineligible for inclusion.

Non-diabetic individuals with a hemoglobin A_1c_ (HbA1c) below 39 mmol/mol (5.7%) and fasting plasma glucose below 6.1 mmol/L at time of screening were eligible for inclusion as NG controls. Individuals with a history of gestational diabetes were ineligible for inclusion as NG controls.

Individuals diagnosed with T2D according to the World Health Organization criteria within 5 years, with an estimated glomerular filtration rate above 60 mL/min, and an HbA1c level below 75 mmol/mol (9.0%) within 3 months of inclusion were eligible for inclusion. Individuals with known monogenic or autoimmune diabetes were ineligible for inclusion. Individuals whose metformin-treatment status could not be ascertained were also excluded.

After applying the inclusion/exclusion criteria above, we included 279 Danes—138 NG controls and 141 with T2D.

Volunteers were examined in the morning following an 8-h overnight fast. Participants were weighed on an electronic scale (TANITA BC-420MA, Tanita Corporation of America, USA) without shoes, dressed in light indoor clothing or underwear after having emptied their bladder. Height was measured to the nearest 0.5 cm without shoes, using a wall-mounted stadiometer (ADE MZ10023, ADE, Hamburg, Germany). BMI was calculated as weight in kilograms divided by the square of height in meters. Waist and hip circumference were measured to the nearest 1 cm in erect position midway between the iliac crest and the lower costal margin, and at the level of the pubic symphysis, respectively. Body composition was assessed using bioelectric impedance analysis (TANITA BC-420MA, USA). Blood pressure was recorded as the mean of duplicate measurements on the non-dominant arm, in reclined position after a 5-min rest.

Blood was collected by puncture of the antecubital vein in the morning after an 8-h overnight fast. Plasma glucose concentration was analyzed by the glucose oxidase method using a colorimetric slide test on a Vitros 5600 system (Ortho Clinical Diagnostics, USA; CV 6.1%). Plasma triglyceride (TG), total cholesterol (TC), and high-density lipoprotein (HDL) were analyzed on a Vitros 5600 system (CV 14.6%, 11.6%, and 17.0%, respectively). Very-low-density lipoprotein (VLDL) was calculated as VLDL = 0.45 × TG. Low-density lipoprotein was calculated as LDL = TC-HDL-VLDL. HbA1c was analyzed by high-performance liquid chromatography (HPLC) on a TOSOH G8 system (Tosoh Bioscience, San Francisco, CA USA, CV 7.2%).

#### Indian sub-study

Two hundred ninety-four Indian volunteers, 137 NG and 157 with type 2 diabetes mellitus, were selected from Dr. Mohans’ Diabetes Specialities Centre, a tertiary care center for diabetes at Chennai, India. The control individuals were selected from an ongoing population-based epidemiological study at Chennai. All individuals were of South Indian (Dravidian) ancestry aged 35 to 65 years, and their BMI ranged from 15.6 to 50.5 kg/m^2^. Individuals suffering from chronic, severe ailments (such as cancer and tuberculosis); those who were pregnant; and those who had used medications such as dipeptidyl peptidase-4 inhibitors, acarbose, glucagon-like peptide-1 receptor agonists, and orlistat were excluded from the study. T2D was diagnosed if the venous plasma glucose 2 h after an oral glucose load and/or the fasting plasma glucose levels were ≥ 7.0 mmol/L. History of diabetes was obtained through self-report. This was then checked against medical records for validity, which helped to define the date and year of diagnosis.

Weight, height, and waist circumference were obtained by trained data collectors using standardized methods. Participants were weighed on an electronic scale without shoes and were asked to wear light clothing, and weight was recorded to the nearest 0.5 kg (TANITA BC-554). Height was measured to the nearest 0.5 cm without shoes, using a stadiometer (SECA 213). Waist and hip circumference were measured to the nearest cm in erect position—waist was measured at the smallest horizontal girth between the coastal margin and the iliac crest and the hip was measured at the greatest circumference at the level of greater trochanters (widest portion of the hip). BMI was calculated as weight (kg) divided by height (m) squared. Blood pressure was recorded from the right arm in a sitting position to the nearest 2 mmHg with a mercury sphygmomanometer (Diamond Deluxe BP apparatus, Pune, India). Two readings were taken 5 min apart, and the mean of the two was taken as the blood pressure. Blood for biochemical analysis was drawn in the morning following an 8-h overnight fast. All biochemical assays including measurement of fasting plasma glucose (hexokinase method), serum cholesterol (cholesterol oxidase–peroxidase–amidopyrine method), serum triglycerides (glycerol phosphate oxidase–peroxidase–amidopyrine method) and, HDL cholesterol (direct method–polyethylene glycol–pretreated enzymes) were measured using Hitachi-912 Autoanalyzer (Hitachi, Mannheim, Germany). Low density lipoprotein (LDL) cholesterol was calculated using the Friedewald formula. HbA1c was measured by high-performance liquid chromatography using the Variant machine (Bio-Rad, Hercules, California, USA). In NG controls, glucose tolerance status was determined from a standard 75 g 2-h oral glucose tolerance test (OGTT). Only individuals with fasting plasma glucose < 6.1 mmol/L and 2-h post-glucose value < 7.8 mmol/L were included in the group of NG healthy controls. C-peptide in plasma was measured using DAKO C-peptide ELISA kit (Dako, Denmark). All biochemistry measurements were performed in the laboratory at the study site which is certified by the College of American Pathologists (Northfield, IL) (No. 7214031) and the National Accreditation Board for Testing and Calibration of Laboratories (New Delhi, India) (M0226).

### Quantification of inflammatory markers

Serum concentration of high-sensitive C-reactive protein (hs-CRP) was measured using a particle enhanced immunoturbidimetric assay with AU680 Clinical Chemistry Analyzer (Beckman Coulter Inc., USA) as per manufacturer’s instructions. Absorbance change (570 nm), with the magnitude of the change being proportional to the quantity of CRP in the sample, was used as the final readout. Actual concentration was then determined by interpolation from a calibration curve prepared from calibrators of known concentration. The intra- and inter-assay precisions were 4.2% and 7.1%, respectively.

Serum MCP-1 levels were measured by AlphaLISA assay (AL509C; Perkin Elmer) as per the manufacturer specifications. The AlphaLISA signal reporting MCP-1 level in the samples was detected at 680-nm excitation and 615-nm emission using an EnSpire Multimode Plate Reader. A standard curve was generated by plotting the AlphaLISA counts versus the concentration of analyte. Raw counts from the experiment were exported using EnSpire Manager (version 4.13.3005.1482). Data were analyzed using nonlinear regression with a 4-parameter logistic equation and MCP-1 levels. The lower limit of detection of the assay sensitivity was 3.8 pg/mL.

A panel of serum cytokines (IL10, IL13, IL17A, IL1β, IL23, IL6, and TNFα) were measured by custom multiplex immunoassay (Human High Sensitivity T Cell multiplex kit: HSTCMAG-28SK-7, Millipore, Billerica, MA, USA) according to the manufacturer’s instructions. Data were acquired on a validated and calibrated Luminex 200 system (Millipore, Billerica, MA, USA). Raw data (mean fluorescence intensities) were captured using the Luminex xPONENT software (v.3.1) and concentrations of immune biomarkers in each sample were interpolated from standard curves using a five-parameter, weighted, logistic regression curve equation in Milliplex Analyst software (v.5.1). For each assay, the curve was derived from various concentrations of the cytokine standards assayed in the same manner as test samples. The lower limits of detection for specific analytes ranged from 0.12 to 2.91 pg/mL based on the manufacturer’s specifications. The methodological details including assay method and precision are available at the manufacturer’s website (www.merckmillipore.com).

LPS-binding protein (LBP) concentrations were measured in diluted serum samples using the sandwich ELISA kit (Human LBP, HK315-02, Hycult Biotech, Uden, The Netherlands) according to the manufacturer’s instructions. The standard curve was created by six-fold serial dilution of a 50 ng/ml standard solution in duplicate. Measurement of LBP levels was performed at 450 nm using EnSpire Multimode Plate Reader. Data were exported using EnSpire Manager software and quantified by standard curve using Graphpad Prism statistical software (v.6). The intra- and inter-assay variability were less than 11.5% and 8.5%, respectively.

Intestinal alkaline phosphatase (IAP) activity was measured in serum samples with a SensoLyte pNPP Alkaline Phosphatase Assay Kit (#71230, AnaSpec, Fremont, CA, USA) according to the manufacturer’s recommendations. Alkaline phosphatase (AP) activity measured in the presence 100 mM L-Phenylalanine (IAP inhibitor) was subtracted from total AP activity to derive levels of serum IAP activity.

### Fecal DNA extraction, library preparation, and sequencing

Fecal samples were collected by the participants following standardized procedures, including home sampling with immediate freezing at − 18 °C in a home freezer and transfer in an insulating polystyrene container with dry ice or cooling elements for final storage at − 80 °C within 48 h.

Microbial genomic DNA was extracted from 200 mg of feces according to a previously published protocol [10]. In brief, samples were chemically lysed by Guanidine Thiocyanate and N-Lauryl sarcosine followed by physical lysis which includes the incubation of samples at 70 °C for 1 h. Samples were then mechanically lysed by bead beating, and the debris, proteins, and aromatic compounds were eliminated using polyvinylpyrrolidone, RNA removed using RNase and ethanol used for the precipitation of purified DNA. Finally, DNA was dissolved in 200 μL TE Buffer and stored at − 80 °C. The concentration and integrity of extracted DNA were estimated by spectrophotometry (NanoDrop, Thermo Fisher Scientific Inc., USA) and agarose gel electrophoresis, respectively.

The variable regions (V1–V5) of the 16S rRNA gene were amplified using 27F (C1) and 926R (C5) primers in 50 μL reaction volume using 0.1 ng of fecal DNA. We used 5–6 nucleotide long barcodes in the reverse primer to label the amplicon of each sample. The PCR-amplified products (950-bp) were gel purified using QIAquick gel elution kit (Qiagen, Germany). The quality of the DNA library was monitored using the High sensitivity DNA chip compatible to 2100 Bioanalyzer (Agilent, USA). Library quantitation was done using PicoGreen dye in QubitFluorometer (Invitrogen, USA). Sequencing of the equimolar libraries was performed on a 454 GS FLX+ pyrosequencer platform (Roche, USA) in two different regions in one picotiter plate, at the Centre for Human Microbial Ecology at the Translational Health Science and Technology Institute (Faridabad, India). Sequence reads obtained in FASTQ format were evaluated by FASTQC [11] using default parameters.

### Sequence data processing

We ensured that at least 5000 high-quality reads (Phred score > 20) were obtained from each sample. V-Xtractor [12] version 2.0 was used to extract the V3–V5 region from the sequenced reads. Reads covering the complete V3–V5 region were kept. OTU picking was performed using the “open reference OTU picking” approach as implemented in the QIIME pipeline [13] (version 1.9.1). We used Greengenes version 13_8 as the reference OTU database, clustering at 97% identity [14], while UCLUST [15] (v 1.2.22) was used as the OTU picking method, using the default parameters. Finally, OTUs containing < 0.002% of the total number of high-quality reads sequenced were removed. A final OTU abundance table with a total of 1895 OTUs was considered for downstream analyses. As the Greengenes database has not been updated since 2013, taxonomy assignment was performed with the RDP classifier as implemented in dada2 package [16] considering SILVA database [17] version 132 as a reference. For more details, please see Additional file 1.

Microbial functional profiles of the microbiome samples were predicted from the respective 16S taxonomic profiles using the PICRUSt [18] software version 1.1.0 (using Greengenes annotation, as it is required by PICRUSt). The results of PICRUSt were curated depicting the relative abundance of KEGG functional modules. The eukaryotic modules were removed before downstream analyses, following the “removal of eukaryotic functions” strategy implemented in Vikodak [19], another tool for predicting the functional potential of the microbial community. As they quantify the potential for these functions encoded in the genomes but not their expression, we refer to them as “functional potential”.

Downstream analysis of the taxonomic and functional profiles was performed in *R*, using *phyloseq* package [20] version 1.24.2. OTU level abundances were appropriately cumulated at higher levels of taxonomic hierarchy using phyloseq, as required for subsequent analysis steps.

### Statistical analysis

Statistical analyses were performed using *R* v.3.4.1 (www.r-project.org).

#### Clinical characteristics

Continuous variables were compared across country and diabetes status by ANOVA with Tukey’s adjustment for multiple contrast. Analysis of covariance was applied to adjust comparisons of waist circumference and waist-to-hip ratio for effects of sex and BMI. Non-normally distributed variables were logarithmically transformed prior to analyses.

#### Microbiome composition and diversity

Sequencing depth differed between samples from the two study populations (Additional file 2: Figure S1). For the alpha-diversity measures, samples were rarefied to an equal sequencing depth of 7000 reads. Differences in alpha-diversity indices (Shannon index and richness) between countries and metformin treatment groups were determined using ANOVA and *t* test on the rarefied read counts.

Differences in community structure were assessed using Bray–Curtis dissimilarity on relative abundances (derived from non-rarefied abundances) as the beta-diversity measure. To estimate the effect of factors on the microbiome composition, we used permutational multivariate analysis of variance implemented in the *adonis* function of the *vegan* package [21] version 2.5.2. For the analysis of differentially abundant taxa (at species, genus, and phylum levels) and functions (at KEGG module level) using non-rarefied read counts, we used the negative binomial Wald test as implemented in the *DESeq2* package version 1.20.0 [22]. Multiple test correction was performed using the Benjamini–Hochberg method considering a false discovery rate (FDR) < 5%. None of the taxa or functional categories was filtered during beta diversity analysis or differential abundance analysis. In the differential abundance analysis, gut microbiome variation between T2D and NG cohorts were adjusted for both metformin and country on the global analysis, and the effect of metformin treatment in the gut microbiome is adjusted for country when necessary. Additionally, we adjusted for gender, BMI, age, sulfonyl urea, statins, and proton pump inhibitors to verify whether they confound the results. We adjusted for these additional factors one by one and took the features that were significantly different across all tests (Additional file 2: Figure S2-S5). The analysis was conducted individually for each parameter in this way because simultaneously adjusting for all these factors led to the method not converging.

Differential prevalence analysis was performed using Fisher’s exact test. Benjamini–Hochberg method considering a false discovery rate (FDR) < 5% was used for multiple test correction. To determine the association between *Christensenellaceae* family and *Oscillospira* genus with visceral fat, we used the Spearman correlation test as it is implemented by the cor.test function in R.

## Results

Clinical characteristics of the Danish and Indians’ samples are presented in Additional file 3: Table S1 and discussed in Additional file 1. In short, when compared with Indian study participants, Danish study participants are older and have higher waist circumference, elevated blood pressure, and elevated circulating lipid concentrations. Microbiome datasets of Danish normoglycemic (NG) and type 2 diabetes (T2D) patients have an average sequencing depth of 16,792 reads/sample and 16,628 reads/sample respectively (Additional file 2: Figure S1A). In the case of the Indian microbiome datasets, the NG group has an average depth of 19,429 reads/sample and the T2D group has 19,418 reads/sample. Within each population, NG and T2D subgroups did not differ in sequencing depth (Additional file 2: Figure S1B).

### Different gut microbial community structures in the Danish and Indian population samples

**Fig. 1:**
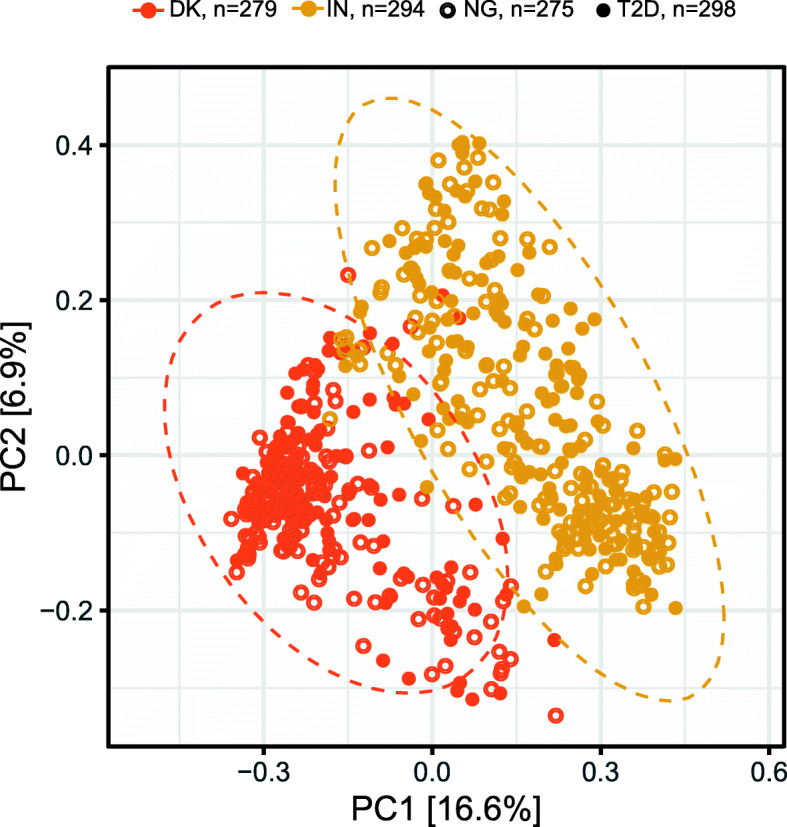
Country of origin is the main driver of gut microbial community structure. Danish and Indian microbiota profiles are clearly separated at OTU level in the principal coordinate analysis using Bray–Curtis dissimilarity measure. Country of origin also explains 12% of the beta-diversity variation (*p* < 0.001, PERMANOVA test). Ellipses represent 95% confidence intervals for the Danish and Indian gut microbiota profiles. Gut microbiota compositions do not separate based on T2D status. DK, Denmark; IN, India; NG, normoglycemic controls; T2D, type 2 diabetes

Multiple factors that might affect the gut microbiota, such as genetics, lifestyle, and environment, are different between the two study populations. Therefore, we searched for differences in the gut microbiota composition between the Danish and Indian population samples, using alpha and beta diversity measures. Analyses of alpha diversity indices, such as OTU richness and Shannon index (Additional file 2: Figure S6), demonstrate that the microbial composition of the Indian study sample is significantly less diverse compared to the Danish study population (*p* < 0.05, ANOVA). Principal coordinate analysis (PCoA) shows a clear separation between the Danish and Indian gut microbiota, demonstrating that the country of origin significantly influences the gut microbiota composition, explaining 12% of the total variation (*p* < 0.001, PERMANOVA) (Fig. 1). Gut microbiota compositions do not separate based on T2D status (Fig. 1), even when we analyze the Danish and Indian samples separately (Additional file 2: Figure S7).

We then looked for individual taxa that were differentially abundant between the two populations using negative binomial regression and correcting for multiple testing using the Benjamini–Hochberg method. A large fraction of the taxa—31 out of 73 families, 97 out of 203 genera and 798 out of 1897 OTUs (Additional file 3: Tables S2 a, b, c)—are differentially abundant between Danes and Indians (*p*_FDR_ < 0.05). We show that 16 families are enriched in the Danish sample, including *Bacteroidaceae*, *Christensenellaceae*, *Verrucomicrobiaceae* (family containing *Akkermansia muciniphila*; 15-fold increase), *Rikenellaceae*, and *Desulfovibrionaceae*; and 15 families are enriched in the Indian sample, including *Lactobacillaceae* (45-fold increase), *Leuconostocaceae*, *Burkholderiaceae*, and *Prevotellaceae*.

At genus level, we find in the Indian population an enrichment of *Prevotella* group 9 (the genus that includes *Prevotella copri*), *Megasphaera* and *Lactobacillus*, and in the Danish population a higher abundance of *Akkermansia*, *Alistipes*, and *Bacteroides* (Additional file 2: Figure S8). Some genera are almost exclusively found in one of the population samples. For instance, *Anaerotruncus* is present in 56% of the Danish samples and only in 3% of the Indians (*p*_FDR_ < 0.05, Fisher’s exact test); and *Achromobacter* is present in 47% of the Indian samples and completely absent in the Danish samples (*p*_FDR_ < 0.05, Fisher’s exact test).

The genus level signatures are mirrored in the OTU level differences in relative abundance. The abundance of 11 OTUs from *Megasphaera*, 36 OTUs from *Prevotella* group 9, 8 OTUs from *Lactobacillus* (including two OTUs from *Lactobacillus ruminis*), 28 OTUs from *Agathobacter*, 12 OTUs from *Catenibacterium*, 12 OTUs from *Collinsella*, and 16 OTUs from *Dorea*, respectively, are enriched in the Indian sample. While the abundance of 39 OTUs from *Blautia* is enriched in the Indian sample, only one is enriched in the Danish sample. Conversely, 42 *Bacteroides* OTUs are enriched in the Danish sample, and only one is enriched in the Indian sample. Two OTUs from *Akkermansia* including *Akkermansia muciniphila,* a prominent mucin degrader associated with host metabolic health*,* are enriched in the Danish study population, whereas there is no enrichment of OTUs from *Verrucomicrobia* in the Indian study population. Interestingly, 15 OTUs from the *Christensenellaceae* family, previously reported to be influenced by host genetics [23], are enriched in the Danish population. Furthermore, abundance of *Christensenellaceae* also inversely correlates with visceral fat in the Danish population (based on spearman correlation with waist-to-hip ratio adjusted for BMI, *r* = − 0.21, *p* = 4.3 × 10^−6^, and waist circumference adjusted for BMI, *r* = − 0.22, *p* = 0.0003) whereas there is no significant association in the Indian individuals (Additional file 2: Figure S9). *Christensenellaceae* has also been inversely associated with visceral fat in a UK population [24], suggesting that this correlation may only apply to western populations.

We also observe that, within some genera present in both Indians and Danes, different OTUs are enriched in the two populations, alluding to population-specific species or strains of commensal gut microbiota. For instance, 23 *Faecalibacterium* OTUs are enriched in the Danish sample, whereas 28 others are enriched in the Indian sample.

### Global and study population-specific signatures of type 2 diabetes at levels of microbial taxonomy and functional potential

**Fig. 2:**
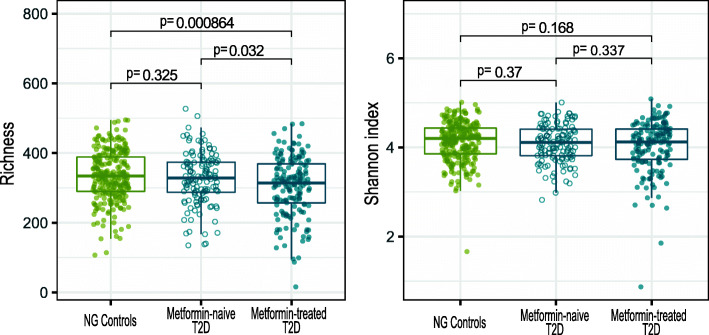
Metformin alters gut microbial richness in combined groups of Danes and Indians. Microbial richness was significantly reduced in metformin-treated T2D patients (*n* = 166), when compared to NG controls (*n* = 275) or metformin-naive T2D patients (*n* = 132). Microbial richness did not significantly differ between NG controls and metformin-naive T2D patients. These differences were not reproduced when looking at Shannon index. Danish and Indians samples were included in the analysis*.* NG, normoglycemic controls; T2D, type 2 diabetes

Microbial richness is significantly decreased in metformin-treated T2D patients compared with metformin-naive T2D patients (*p* = 0.032, *t* test; Fig. 2) and NG controls (*p* = 8.6·10^−4^, t-test; Fig. 2). With Shannon diversity index, there is a trend for metformin-treated T2D patients to exhibit lower diversity than NG controls (*p* = 0.168, *t* test). Both alpha diversity indices show that there was no significant difference between NG controls and metformin-naive T2D patients, highlighting the negative effect of metformin treatment on gut microbiota richness. Within the individual population samples, we reproduce the reduced microbial richness in metformin-treated T2D individuals compared to NG controls, but the Shannon diversity index was not significantly different between any of the groups (Additional file 2: Figure S10).

Given the strong study population effect on the gut microbiota composition, we adjusted for it in all downstream analyses. We looked for T2D-associated differentially abundant taxa in the combined group of T2D patients (*n* = 298) compared with all NG controls (*n* = 275) using negative binomial regression. After correcting for multiple testing, we identify 33 differentially abundant OTUs and 5 differentially abundant genera (*p*_FDR_ < 0.05; Additional file 3: Table S3).

**Fig. 3:**
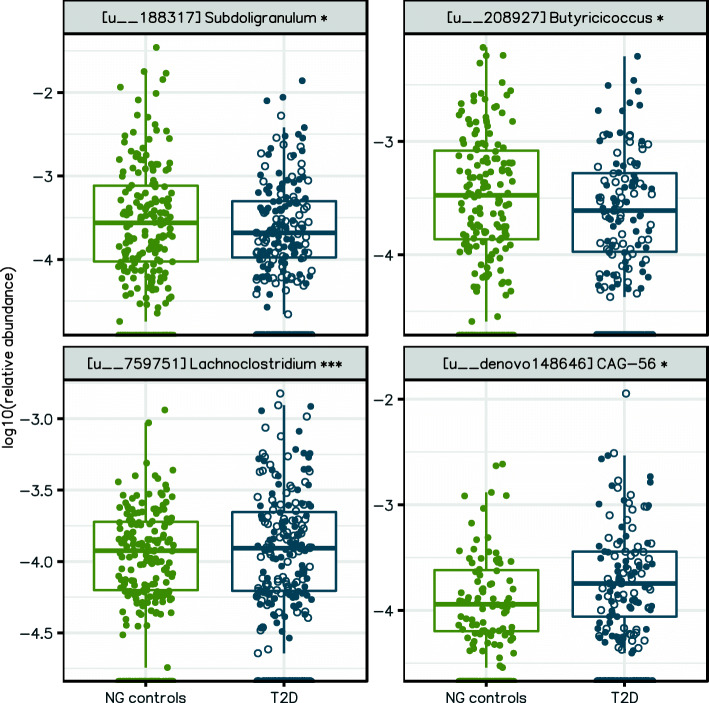
OTUs are differentially abundant in the microbiota from the combined Danish-Indian group of T2D patients after controlling for confounding factors. Relative abundances of differentially abundant OTUs are shown in log scale. Among T2D patients, metformin-treated individuals are represented by filled circles and metformin-naive individuals are represented by hollow circles. Danish and Indians’ samples are included in the analysis (also see Additional file 2: Figure S11 for separate visualizations for the countries). These four OTUs are consistently identified from six different analyses where country and metformin were always adjusted for, while each additional factor (BMI, age, gender, usage of sulfonyl urea, statins, and proton pump inhibitors) was adjusted for one by one. NG, normoglycemic controls (*n* = 275); T2D, type 2 diabetes (*n* = 298)

More than half of the T2D patients in our study population were treated with metformin (Additional file 3: Table S1), which has previously been shown to affect the gut microbiota [4, 5]. Additionally, many patients were also treated with sulfonylurea (used by > 30% of Indian diabetics), proton pump inhibitors (frequently used and with known effects on the gut microbiota), and statins. Finally, anthropometric characteristics including age, gender, and BMI may also confound the T2D-association analysis. Therefore, we repeated the T2D-associated differential abundance analysis while additionally controlling for these potential confounding factors. Simultaneously controlling for all the factors failed due to convergence issues in the analysis software. To solve this, we tested for the confounding factors one by one and identified T2D-associated differentially abundant taxa that were not confounded by any of the factors. Being a known major confounding factor, metformin treatment (metformin-naive T2D, *n* = 132; and metformin-treated T2D, *n* = 166) was always controlled for in addition to country of origin. Only 4 out of 33 OTUs (12.1%) and 2 out of 5 genera remain differentially abundant (*p*_FDR_ < 0.05; Additional file 3: Table S4; Fig. 3, Additional file 2: Figure S2 a-b), highlighting the importance of adjustment for the confounding factors. As an alternative to adjustment for study population, we also performed a meta-analysis of metformin-free T2D signatures from the two study populations. While none of the OTUs were statistically significant (*p*_FDR_ < 0.05; see Additional file 3: Table S5a), two out of the three near-significant OTUs (*p*_FDR_ < 0.10) in the meta-analysis were also discovered in our original analysis. Hence, we used the original results rather than meta-analysis results as global T2D signatures. In the combined Danish-Indian group, T2D patients have increased abundance of two OTUs from the *Lachnospiraceae* family (Fig. 3, Additional file 3: Table S4a). Similarly, T2D patients also have decreased abundance of two OTUs from the *Ruminococcaceae* family (*Subdoligranulum* and *Butyricicoccus* genera). At genus level, *Lachnoclostridium* is enriched in T2D microbiota whereas *Anaerosporobacter* is depleted in T2D microbiota (Additional file 3: Table S4b).

Similar analyses were conducted within the individual study populations. In the Danish study sample (metformin- naive T2D, *n* = 61; metformin-treated T2D, *n* = 80; and NG controls, *n* = 138), the genus *Lachnoclostridium* is enriched in T2D patients (*p*_FDR_ < 0.05; Additional file 3: Table S6). When we repeated this analysis in the Indian study sample (metformin-naive T2D, *n* = 71; metformin-treated T2D, *n* = 86; and NG controls, *n* = 137), none of the OTUs or the genera shows differential abundance based on our statistical criteria.

**Fig. 4:**
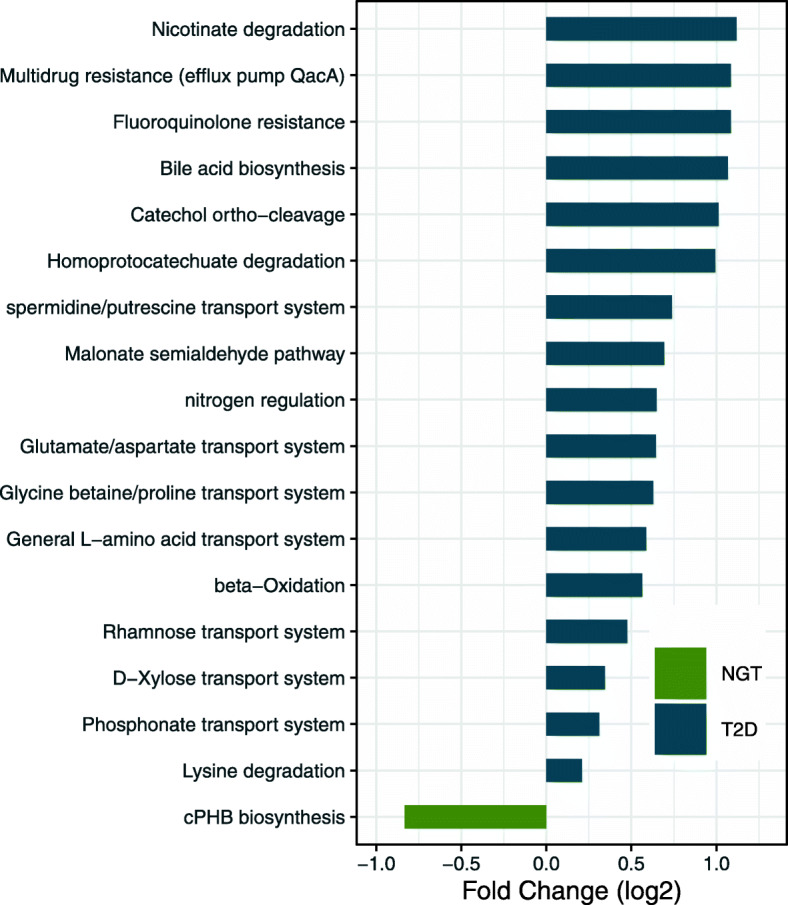
Microbial functional modules are differentially abundant in the microbiota of the combined Danish-Indian group of T2D patients after controlling for confounding factors. Fold change of differentially abundant functional modules are shown in log scale. Both Danish and Indians’ samples were included in the analysis. These modules are consistently identified from six different analyses where country and metformin were always adjusted for, while each additional factor (BMI, age, gender, usage of sulfonyl urea, statins, and proton pump inhibitors) was adjusted for one by one. NG, normoglycemic controls (*n* = 275); T2D, type 2 diabetes (*n* = 298)

We next investigated the signatures of T2D in the functional potential of the gut microbiome. For this, we used the microbial functional profiles imputed by PICRUSt [18]. Following the same methodology as for taxonomic analysis (using negative binomial regression, adjusting for confounding factors and correcting for multiple testing using the Benjamini–Hochberg method), we find that 18 out of 481 KEGG modules are differentially abundant between the combined group of T2D patients and the combined group of NG controls (Fig. 4, Additional file 2: Figure S3, Additional file 3: Table S7). Meta-analysis of metformin-adjusted functional profile signatures revealed 17 differentially abundant KEGG modules (*p*_FDR_ < 0.05; Additional file 3: Table S5b), 9 out of which were also identified in our original analysis. Therefore, we considered the original 18 KEGG modules as global T2D signatures.

T2D patients have a significant enrichment of predicted environmental information-processing functions (10 KEGG modules); with 7 modules from ABC transporters, 2 modules from two-component regulatory systems and 1 module related to drug resistance mechanisms. The modules from the ABC transporters are distributed between three transporter systems: monosaccharide transporters (Rhamnose and D-Xylose), phosphate and amino acid transporters (Glutamate/aspartate, General L-amino acid and Phosphonate transporters) and mineral and organic ion transporters (spermidine/putrescine and Glycine betaine/proline). Further predicted enriched modules belong to carbohydrate and lipid metabolism, and nucleotide and amino acid metabolism.

In the Danish population, 5 predicted metabolic modules are enriched in the T2D patients (Additional file 3: Table S8). Among these, two are also found in the combined group analysis. Three of these modules are associated with saccharide transport systems and one with thiamine transport. In the Indian population, none of the predicted metabolic modules shows differential enrichment.

### Effect of metformin on gut microbiota of patients with T2D

**Fig. 5:**
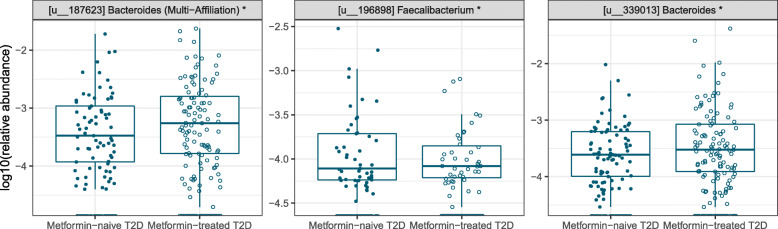
OTUs in the gut microbiota of the combined Danish-Indian group of T2D patients are affected by metformin treatment. Relative abundances of differentially abundant OTUs are shown in log scale. Both Danish and Indians’ samples were included in the analysis. Comparisons were made between T2D metformin-treated (*n* = 166) and T2D metformin-naive (*n* = 132). These OTUs are consistently identified from six different analyses where country was always adjusted for, while each additional factor (BMI, age, gender, usage of sulfonyl urea, statins, and proton pump inhibitors) was adjusted for one by one. T2D, type 2 diabetes

Considering the strong confounding effect of metformin in our analysis, we investigated the effect of metformin treatment on the gut microbiota of our T2D patients. We compared metformin-treated T2D (*n* = 166) and metformin-naive T2D (*n* = 132) patients from both populations after controlling for study effect and other confounding factors (Additional file 3: Table S9, Additional file 2: Figure S4). Here we identify 3 differentially abundant OTUs using negative binomial regression (p_FDR_ < 0.05; Fig. 5). Metformin-treated T2D patients have an enrichment of 2 OTUs from *Bacteroides* and lower abundance of one OTU from *Faecalibacterium.* At the genus level none of the genera shows differential enrichment based on our statistical criteria. When we repeated this analysis for the individual populations, the genus *Escherichia*/*Shigella* is enriched in Danish metformin-treated T2D patients (p_FDR_ < 0.05; Additional file 3: Table S10). In the Indian study sample, one OTU from *Lachnoclostridium* is enriched in metformin-treated T2D patients (*p*_FDR_ < 0.05; Additional file 3: Table S11).

When we combine Danish and Indian samples, we do not find any differential abundant KEGG module after controlling for confounding factors (Additional file 2: Figure S4). In the individual study population analysis, we only find metformin signals in the Danish population, where 4 predicted modules are increased in metformin-treated T2D patients, including nitrogen metabolism, drug resistance, fatty acid metabolism, and manganese/zinc/iron transporters (Additional file 3: Table S12).

## Discussion

We present the gut microbial signatures in Danish and South Indian individuals who are highly diverse in their geographic location, ethnicity, and lifestyle including dietary factors. We observe major compositional differences between the Danish and South Indian gut microbiota when analyzing the combined diabetic and nondiabetic subgroups from the two countries. We find enrichment of *Prevotella* group 9 and *Megasphaera* in the Indian sample*,* previously reported as distinctive features of the Indian gut microbiota [25]. Higher relative abundance of *Prevotella* has been associated with higher habitual plant-based fiber intake [26], and *Prevotella* was shown to rapidly decrease in relative abundance in omnivorous subjects when switching to an all animal-based diet [27]. Thus, the enrichment of *Prevotella* in the Indian sample alludes to the dietary differences, as suggested previously [25]. We also observe an enrichment of *Lactobacillus* in Indians, which might be due to higher consumption of fermented foods [25]. On the other hand, we find a higher relative abundance of *Bacteroides* in the Danish population, consistent with previous results for Western countries [28, 29]. *Bacteroides* was shown to rapidly increase in omnivorous subjects when switching to an all animal-based diet [27]. Studies have suggested that the balance between *Bacteroides* and *Prevotella* is determined by the balance between predominantly animal- and plant-based diets, which our results support. We also identify a strong enrichment of bile acid tolerant bacteria enriched in Danish samples, further reinforcing the effect of a more animal-based diet in Danish gut microbiota—11 OTUs from *Alistipes*, 11 OTUs from *Parabacteroides*, and one OTU from *Bilophila wadsworthia*.

Our search for population-specific T2D signatures at the taxonomy level fails in both countries, as none of the OTUs at species level is differentially abundant. This observation suggests that a taxonomic microbiota signal in Indian and Danish T2D patients, if present, is difficult to detect. In combined analysis, T2D patients have decreased abundance of short-chain fatty acid (SCFA) producers from the *Ruminococcaceae* family such as *Subdoligranulum* and butyrate-producing *Butyricicoccus*, consistent with previous results [4]. Only 2 genera (*Anaerosporobacter* and *Lachnoclostridium*) associate with T2D after controlling for the effect of metformin.

Functionally, T2D patient microbiomes have an enrichment mainly in carbohydrate and lipid metabolism; nucleotide and amino acid metabolism; two-component regulatory systems; several transport systems; and drug resistance. Particularly, enrichment of sugar transport systems in T2D patients suggests that this could alter the availability of sugars to the host thereby affecting glucose homeostasis. Furthermore, we also find an increased potential for lysine degradation in T2D patients. These findings are in accordance with previously reported depletion of lysine in serum from T2D patients [30, 31]. Given the limitations of inferring functional potential from 16S rRNA amplicon sequencing data, our findings require future validation using shotgun metagenomics.

With a reasonably balanced proportion of metformin-naive and metformin-treated T2D patients in our combined study sample, we had sufficient statistical power to look for global signatures of metformin in the gut microbiota. Metformin treatment is associated with reduced abundance of *Faecalibacterium*, a SCFA producer widely considered as beneficial. While this is unexpected, a reduction of *Faecalibacterium prausnitzii* has previously been seen in Spanish individuals after a 4-month metformin treatment [32]. The enrichment of *Escherichia*/*Shigella* in metformin-treated T2D patients from the Danish cohort but not in the Indian cohort after adjusting for the effect of BMI, age, gender, and additional medications is consistent with results from our previous multi-cohort analysis of the effect of metformin [4]. In our previous Chinese-Scandinavian study sample, increased abundance of *Escherichia* was observed in Danish and Swedish T2D study participants, but not in the Chinese T2D patients [4], suggesting that this effect may be associated with ethnicity or demography.

Overall, our study design and the number of participants allow us to assess “universal” microbial taxonomic associations with T2D, while controlling for confounding effects due to ethnic variations, effects of T2D treatments, and factors such as gender, BMI, and age. While our study indicates that ethnic signatures overshadow T2D-specific signatures, some of the microbial signatures show a more profound and somewhat universal trend.

## Conclusions

In the present trans-ethnic study, we demonstrate major differences between the Danish and Indian gut microbiota, some of which may relate to differences in demography and dietary practice. By comparing T2D patients and NG individuals, we identify T2D-related microbial changes in taxonomy within the Danish population sample. Such changes are not apparent in the Indian sample potentially due to Indians having more individually diverse intestinal microbial communities putting more demand on study sample size when comparing affected versus non-affected individuals. We also identify gut microbiota changes associated with metformin treatment, which confirms previously known associations. Finally, across the two population samples, we identify gut microbial signatures which associate with T2D that are not confounded by country, medication, or other confounding factors. These signatures are represented by few taxa—4 OTUs and 2 genera—suggesting that identification of gut microbial signatures of treatment-naive T2D is a challenge when applying the 16S rRNA gene amplicon approach with a moderate taxonomic resolution.

## Supplementary Information

**Additional file 1.** Supplementary Material consisting two sections – Supplementary Methods and Supplementary Results (Format: PDF).

**Additional file 2 **Eleven supporting **Figures S1-S11**. A figure caption for each is given within the file (Format: PDF).

**Additional file 3.** Twelve supporting Tables. A table caption for each is given within the file (Format: Excel Spreadsheet).

## Data Availability

Sequence data for all microbiome samples have been submitted to NCBI SRA and are available with SRA accession PRJNA517829 (https://www.ncbi.nlm.nih.gov/bioproject/PRJNA517829/) [33].

## Abbreviations

HbA1c: Glycated hemoglobin
KEGG: Kyoto Encyclopedia of Genes and Genomes
NG: Normoglycemic
OTU: Operational taxonomic unit
PCoA: Principal coordinate analysis
*p*_FDR_: FDR-adjusted *P* value
rRNA: Ribosomal RNA
SCFA: Short-chain fatty acid
T2D: Type 2 diabetes
