# Supplementary material for: Trans-ethnic gut microbiota signatures of type 2 diabetes in Denmark and India
Source: Genome Med. 2021 Mar 3;13:37. doi: 10.1186/s13073-021-00856-4 (PMC7931542; doi:10.1186/s13073-021-00856-4)
Supplement: Supplementary file 1 — Additional file 1. Supplementary Material consisting two sections – Supplementary Methods and Supplementary Results (Format: PDF). [file 13073_2021_856_MOESM1_ESM.docx]

# Supplementary Materials

## Supplementary Methods

This work is a substudy of a large Indo-Danish consortium investigating the gut microbiome in both type 2 diabetes (T2D) and prediabetes. Both the T2D substudy (present study) and the prediabetes substudy (manuscript submitted) use the same individuals as normoglycaemic (NG) controls. The consortium recruited and collected samples from a total of 864 individuals (NG, n=275; T2D, n=300; prediabetics, n=289). To ensure consistency and comparability between the prediabetes substudy and the T2D substudy, we processed all 864 samples consistently using the same molecular biology techniques and computational analysis pipeline.

Considering a minimum sequencing coverage of 5,000 high quality reads per sample, 18,380,379 quality filtered reads (Phred score >20) were obtained from the 864 samples. V-Xtractor^12^ version 2.0 was used to extract the V3-V5 region from the sequenced reads. A total of 17,030,870 quality checked and trimmed reads, which cover the complete V3-V5 region, were considered for the downstream analyses (average sequencing depth of 19,712 ± 7774 reads/sample). OTU picking was performed using the ‘open reference OTU picking’ approach as implemented in the QIIME pipeline^13^ (version 1.9.1). We used Greengenes version 13_8 as the reference OTU database, clustering at 97% identity^14^, while UCLUST^15^ (v 1.2.22) was used as the OTU picking method, using the default parameters. Finally, OTUs containing <0.002 % of the total number of high-quality reads sequenced, were removed. We obtained 1897 OTUs, including 1471 OTUs already catalogued in the Greengenes database, as well as 426 de novo OTUs from the 864 samples. As the Greengenes database has not been updated since 2013, taxonomy assignment was performed with the RDP classifier as implemented in dada2 package^16^ considering SILVA database^17^ version 132 as a reference. A total of 10,372,258 quality-checked and trimmed reads, were assigned to a subset of 573 samples, pertaining to normoglycaemic and type 2 diabetes individuals from Denmark and India (Indians: 137 NG + 157 T2D; Danes: 138 NG + 141 T2D). A final OTU abundance table with a total of 1895 OTUs identified from 573 microbiome samples corresponding to the T2D patients and NG control individuals was considered for downstream analyses.

## Supplementary Results

Clinical characteristics of the Danish and Indians samples are presented in Supplementary table 1. Men and women are equally represented among T2D patients and normoglycaemic controls in the Indian study samples. Conversely, women are overrepresented among normoglycaemic Danes (70%) and underrepresented among Danes with T2D (30%). Danish T2D participants are 10 years older on average than their Indian counterparts (62±7.8 y vs. 51±8.1 y, respectively; p<0.001). When adjusting for sex and BMI, participants with T2D from the two countries have the same amount of body fat (mean difference 0.9 [-0.3; 2.1] %-points; p=0.13). However, but Danish participants have higher waist circumference (mean difference 5.0 [3.5; 6.4] cm; p<0.001) and waist-to-hip ratio (mean difference 0.03 [-0.01; 0.05]; p=0.002) indicating that intra-abdominal fat deposition is more pronounced among Danish diabetics.

Long-term glycaemic control represented by glycated haemoglobin A1c is better among Danish T2D participants than Indians with T2D (6.6±1.1 % [49±11.7 mmol/mol] vs. 7.8±2.0 % [62±21.8 mmol/mol]; p<0.001); but there is no difference between Danish and Indian normoglycaemic controls (5.2±0.3% [33±2.9 mmol/mol] vs. 5.4±0.4 % [36±4.0 mmol/mol]; p=0.26). More than half of the participants with T2D are treated with one or more hypoglycaemic drugs (62% in Denmark and 58% in India). In both countries, metformin is the oral anti-hyperglycaemic agent most frequently used (57% and 55% of Danes and Indians, respectively; p=0.82). Among Danes, dipeptidyl peptidase-4 inhibitors are the second most frequently used blood glucose lowering compound (8% vs. 1% of Indians; p=0.02), whereas sulfonylureas are the second most frequently used antidiabetic drug among Indians (34% vs. 6% of Danes; p<0.001). In addition, 10% of Danish participants with T2D are treated with subcutaneous injection of a glucagon-like peptide-1 receptor agonist and 8% are treated with insulin. In contrast, no Indian T2D participants were treated with injectable medications.

Lipidaemic control represented by fasting plasma levels of total cholesterol and low-density lipoprotein cholesterol is similar in Danish and Indian diabetics (mean difference 0.1 [-0.3; 0.2] mmol/L and 0.2 [-0.1; 0.4] mmol/L, respectively); however, use of statins is more frequent among Danish participants with T2D (57% vs. 13% among Indians; <0.001). Few normoglycaemic participants from either country are treated with statins (4% and 3% in Denmark and India, respectively); interestingly, fasting plasma levels of total cholesterol (mean difference 0.8 [0.6; 1.1] mmol/L; p<0.001) and LDL cholesterol (mean difference 0.5 [0.3; 0.8] mmol/L; p<0.001) are higher in normoglycaemic Danes further indicating that lipidaemia is less pronounced in the Indian population than the Danish population.

Antihypertensive therapy is more commonly prescribed to Danes with T2D than among Indians (56% and 12%, respectively), but systolic blood pressure (SBP) is still higher in Danes than in Indians (mean difference 10.3 [6.3; 14.3] mmHg; p<0.001). Among normoglycaemic participants, SBP is also higher in Danes (mean difference 7.2 [3.0; 11.3] mmHg; p<0.001) further indicating that SBP is lower and hypertension less pronounced in the Indian population in general.

Results from NG and T2D individuals on a panel of eleven fasting serum inflammatory biomarkers are also presented in Supplementary table 1. Among Danes, patients with T2D have significantly (Wilcoxon test, P-adj<0.05) higher levels of serum C-reactive protein (h-CRP), Interleukin 6 (IL6), Tumor Necrosis Factor α (TNFα), and intestinal alkaline phosphatase (IAP) compared with NG individuals. Indian patients with T2D have significantly (Wilcoxon test, P-adj<0.05) higher levels of serum C-reactive protein (h-CRP) compared to NG individuals, while other inflammatory markers show no statistical significance. Interestingly, irrespective of the glycaemic status, the overall levels of a majority of the inflammatory markers (except MCP1, IL13 and IL17A) are considerably higher among Indians compared to Danes implying a state of higher sub-clinical systemic inflammation in Indians.
