# Supplementary material for: Trans-ethnic gut microbiota signatures of type 2 diabetes in Denmark and India
Source: Genome Med. 2021 Mar 3;13:37. doi: 10.1186/s13073-021-00856-4 (PMC7931542; doi:10.1186/s13073-021-00856-4)
Supplement: Supplementary file 2 — Additional file 2 Eleven supporting Figures S1-S11. A figure caption for each is given within the file (Format: PDF). [file 13073_2021_856_MOESM2_ESM.docx]

Supplementary figures


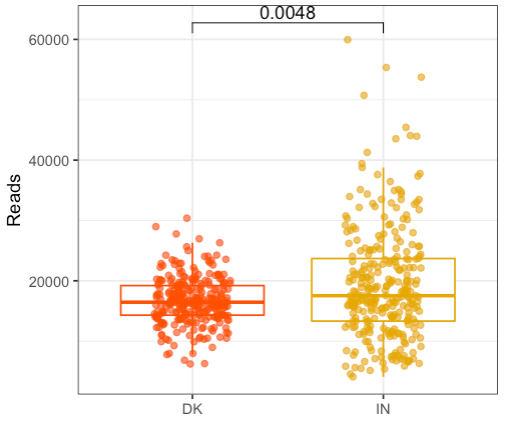

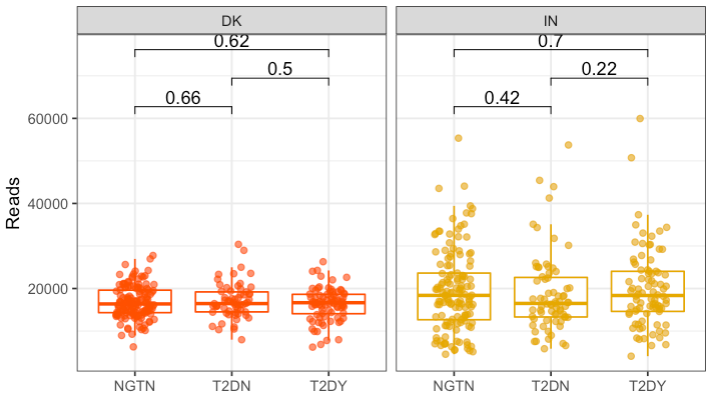


**A**

**B**

**Figure S1**. **A.** Comparison of sequencing depth between Danish (n=279) and Indian gut microbiota (n=294). **B.** Comparison of sequencing depth between health status and metformin treatment within each country. Denmark (NGTN, n=138; metformin-naive T2DN, n=61; metformin-treated T2DY, n=80) and in India (NGTN, n=

**A.** OTUs that are differentially abundant between T2D and NG

**Global**

**DK**

**B.** Genera that are differentially abundant between T2D and NG

**Global**

**DK**

**Figure S2**. **Gut microbial signatures that are differentially abundant between Type 2 diabetes samples and normoglyaceamic controls after controlling for confounding factors.** **A)** Differentially abundant OTUs. **B)** Differentially abundant genera. For global analysis, country and metformin were adjusted for in all analysis, while each additional factor (SU, Statins, PPI, BMI, age and gender) was adjusted for one by one. For country-specific analysis, the procedure was the same except that it was not necessary to adjust for country. Significant features are highlighted in green. DK: Denmark; IN: India; SU: sulfonyl urea; PPI: proton pump inhibitors.

**Global**

**DK**

**IN**

**Figure S3**. **KEGG modules that are differentially abundant between** **Type 2 diabetes samples and normoglyaceamic controls after controlling for confounding factors.** For global analysis, country and metformin were adjusted for in all analysis, while each additional factor (SU, Statins, PPI, BMI, age and gender) was adjusted for one by one. For country-specific analysis, the procedure was the same except that it was not necessary to adjust for country. Significant features are highlighted in green. DK: Denmark; IN: India; SU: sulfonyl urea; PPI: proton pump inhibitors.

**A.** OTUs that are differentially abundant between metformin-naive T2D and metformin-treated T2D

**Global**

**DK**

**IN**

**B.** Genera that are differentially abundant between metformin-naive T2D and metformin-treated T2D

**Global**

**DK**

**IN**

**Figure S4**. **Gut microbial signatures that are differentially abundant between** **metformin-naive T2D and metformin-treated T2D patients after controlling for confounding factors.** **A)** Differentially abundant OTUs. **B)** Differentially abundant genera. For global analysis, country was adjusted for in all analysis, while each additional factor (SU, Statins, PPI, BMI, age and gender) was adjusted for one by one. For country-specific analysis, the procedure was the same except that it was not necessary to adjust for country. Significant features are highlighted in green. DK: Denmark; IN: India; SU: sulfonyl urea; PPI: proton pump inhibitors.

**Global**

**DK**

**Figure S5**. **KEGG modules that are differentially abundant between** **metformin-naive T2D and metformin-treated T2D patients after controlling for confounding factors.** For global analysis, country was adjusted for in all analysis, while each additional factor (SU, Statins, PPI, BMI, age and gender) was adjusted for one by one. For country-specific analysis, the procedure was the same except that it was not necessary to adjust for country. Significant features are highlighted in green. DK: Denmark; IN: India; SU: sulfonyl urea; PPI: proton pump inhibitors.

**
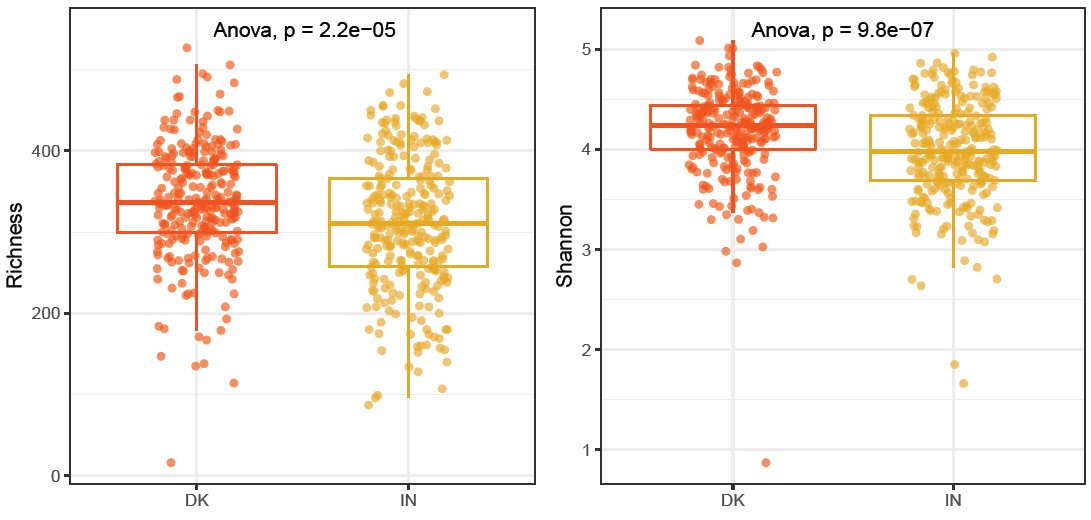
Figure S6**. **Danish gut microbiota (n=279) exhibits higher alpha diversity at OTU level compared to Indian gut microbiota (n=294).** Danish and Indian populations include Type 2 diabetes samples and normoglyaceamic controls for each country. DK: Denmark; IN: India.

**
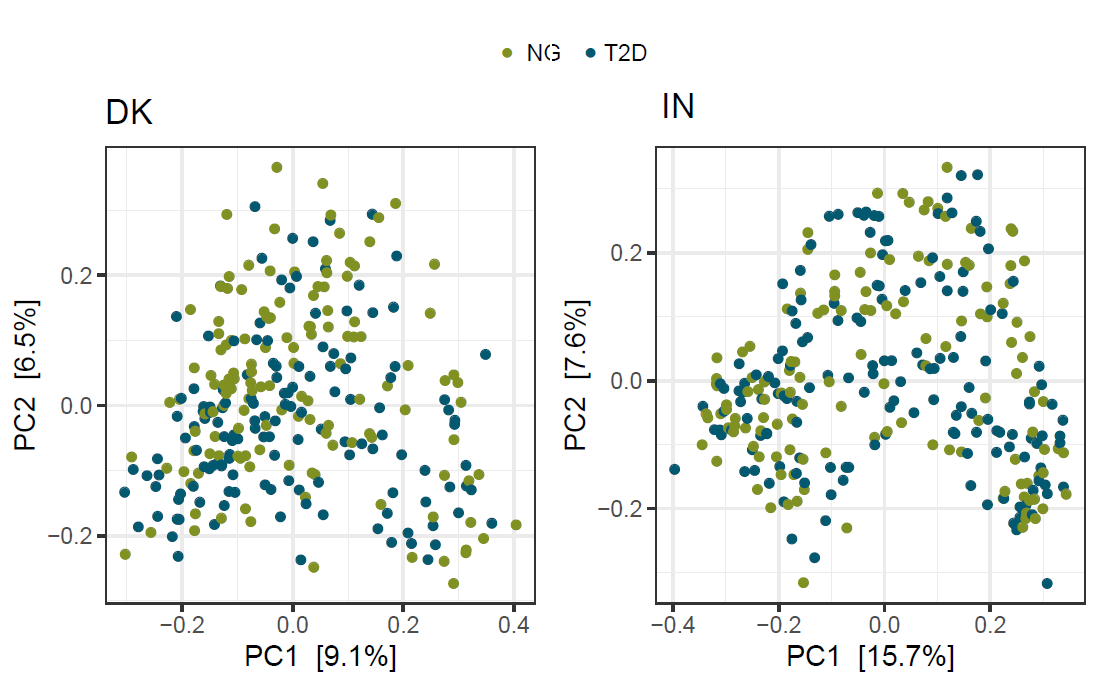
Figure S7**. **Principal coordinate analyses of Danish (DK, n= 279) and Indian (IN, n=294) gut microbiota profiles at OTU level do not reveal a separation of samples based on T2D status**. Bray-Curtis dissimilarity measure is used as beta-diversity measure. NG: normoglycaemic controls; T2D: type 2 diabetes.


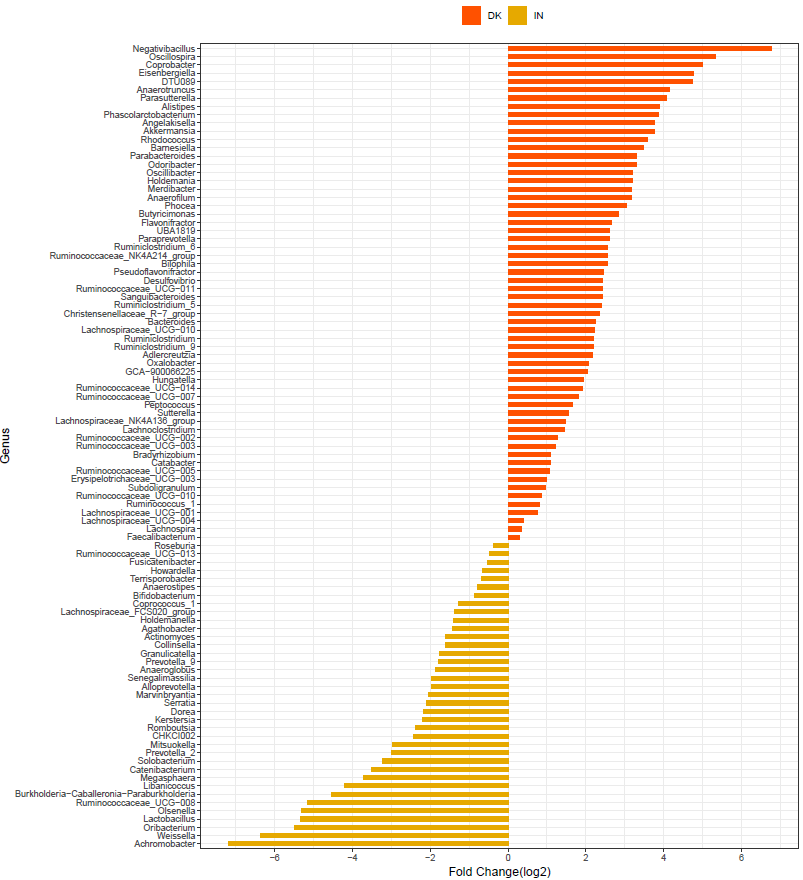


**Figure S8.** **Genera differentially abundant in the gut microbiota from Danish and Indian study participants.** (DK, n=279) and India (IN, n=294).


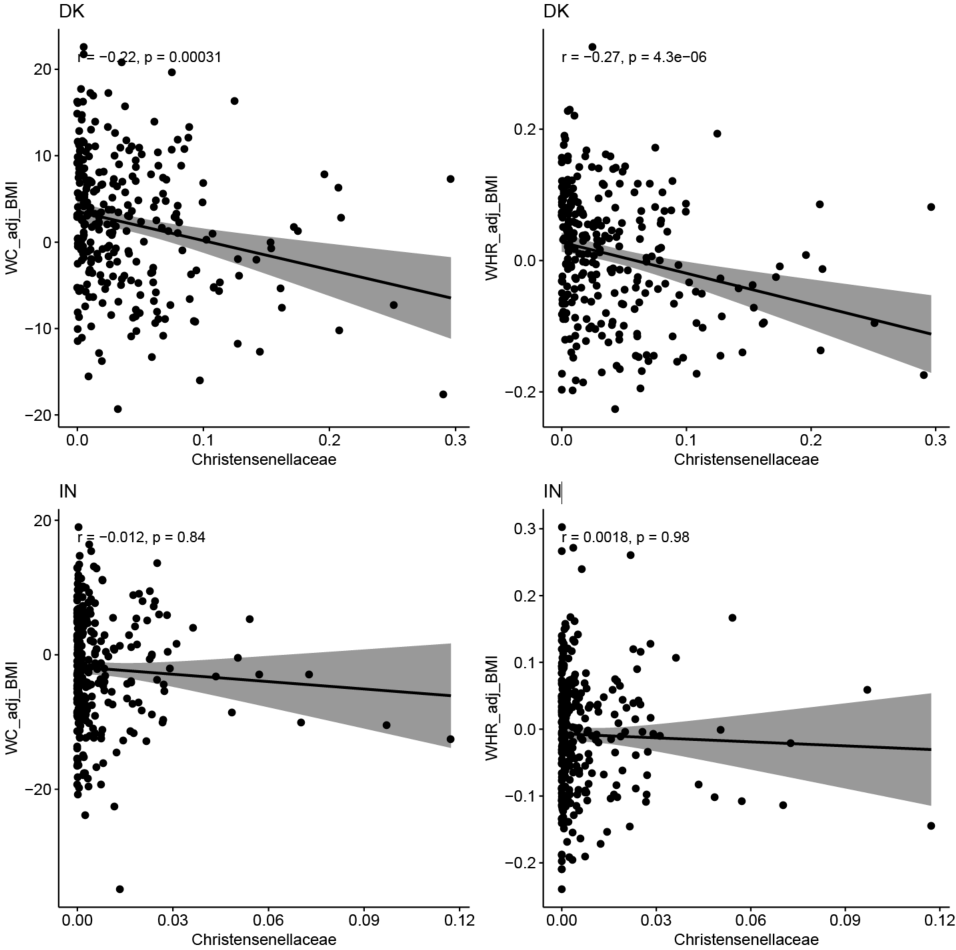


**Figure S9**. **Correlations between relative abundance of Christensenellaceae and two markers of visceral fat: waist circumference adjusted for BMI (WC_adj_BMI) and waist-to-hip ratio adjusted for BMI (WHR_adj_BMI).** DK: Denmark (n=279); IN: India (n=294).

**
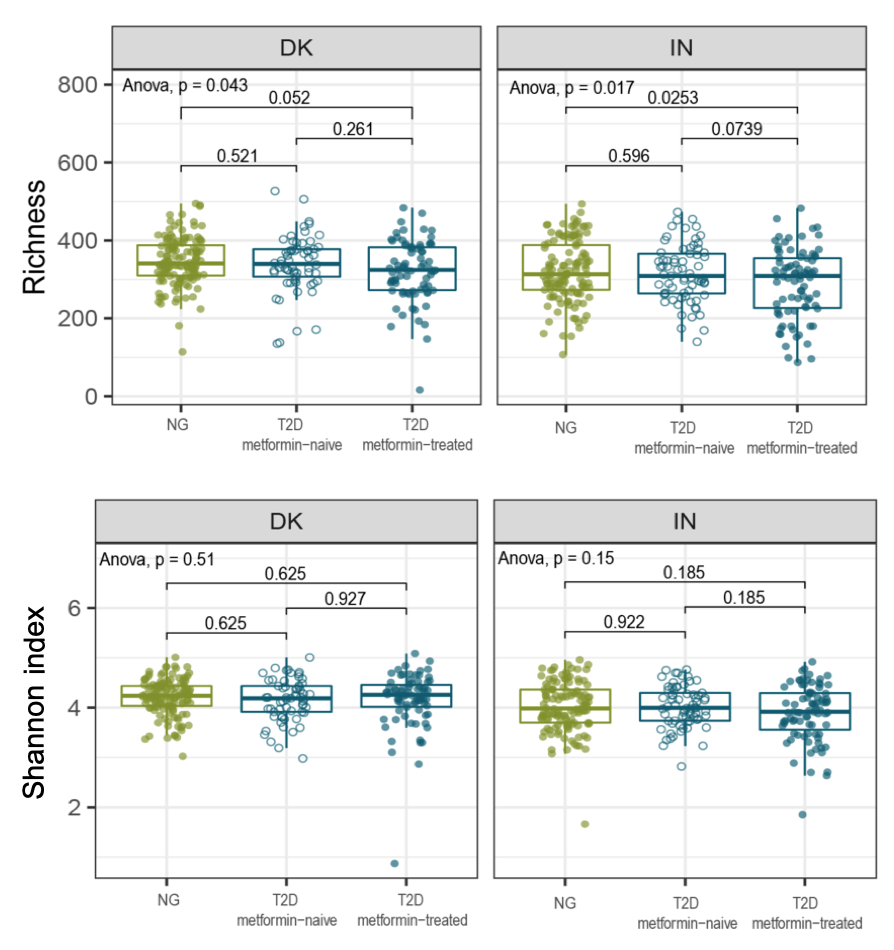
**

**Figure S10**. **Gut microbial alpha-diversity at the OTU level is reduced in metformin-treated T2D patients, both in Denmark (NG, n=138; metformin-naive T2D, n=61; metformin-treated T2D, n=80) and in India (NG, n=137; metformin-naive T2D, n=71, metformin-treated T2D, n=86).** DK: Denmark; IN: India.

******

**Figure S11. The relative abundances of the 4 OTUs differentially abundant in the microbiota from the combined Danish-Indian group of T2D patients after controlling for country, metformin treatment and other covariates are shown in log scale, separated by country.** These four OTUs are consistently identified from six different analysis where country and metformin were always adjusted for, while each additional factor (BMI, age, gender, usage of sulfonyl urea, statins, and proton pump inhibitors) was adjusted for one by one. NG: normoglycaemic controls (n=275); T2D: type 2 diabetes (n=298); DK: Denmark; IN: India.
